# Sand-grinding integrated with coatings for preparation of hydrophobic surface based on Miao silver

**DOI:** 10.1016/j.mex.2026.104159

**Published:** 2026-09-17

**Authors:** Hongyun Zhang, Jian Li, Jinfu Pan, Qiuyan Mo, Yongjun Ma

**Affiliations:** a Engineering Research Center of Micro/nano and Intelligent Manufacturing of Ministry of Education, Kaili University, Kaili, 556011, China; b School of Chemistry & Chemical engineering, Guangxi University, Nanning, 530004, China

**Keywords:** Biomimetic, Micro/nano–structure, Doping, Composite wetting state, Roughness, Solid fraction

## Abstract

Water-proofing, anti-corrosion and anti-tarnishing requirement is currently the key to improvement of Miao silver industry. Inspired by superhydrophobic surface (SHS), we managed to use it as a protecting layer to isolate from air, water vapor, liquid electrolyte possibly around with silver ware; meanwhile also give a guide for other **mental** productions with the same requirement. Considering micro/nanostructure of lotus leaf, water strider’s leg, butterfly wings together surface wax substance, especially for butterfly wings’ scale-like macrostructure with semi-cylinder-like nanostructure on it, a biomimetic semi-cylinder was theoretically put forward to simulate such composite structure. Followed by it, experimentally using sand-grinding integrated with SiO_2_-doped fluorinated modified silane coatings to obtain an SHS with water contact angle (CA) of 162° and CA hysteresis of 4°, micro/nanostructure obtained by coarse/fine sand-grinding in same direction respectively for simulation of butterfly wings’ scale structure (macro size) and cylinder structure (nano size); coatings mainly consist of fluorinated modified silane and nano SiO_2_ grain from doping; thus structure and coating design having some of independent.

Our study emphasizes three aspects below:

1. Theoretically, how to simulate micro/nano structure of butterfly wings by modeling based on the natural and artificial SHS, thus a semi-cylinder model was proposed.
2. Experimentally, how to prepare such an SHS similar to the butterfly wings’ composite structure; thus method of sand-grinding integrated with coatings was configured.
3. How to establish the relationship between microstructure and the roughness, solid fraction, contact angle, adhesion work and spreading coefficient based on the already formed composite wetting state.

## Graphical abstract

**Figure:**
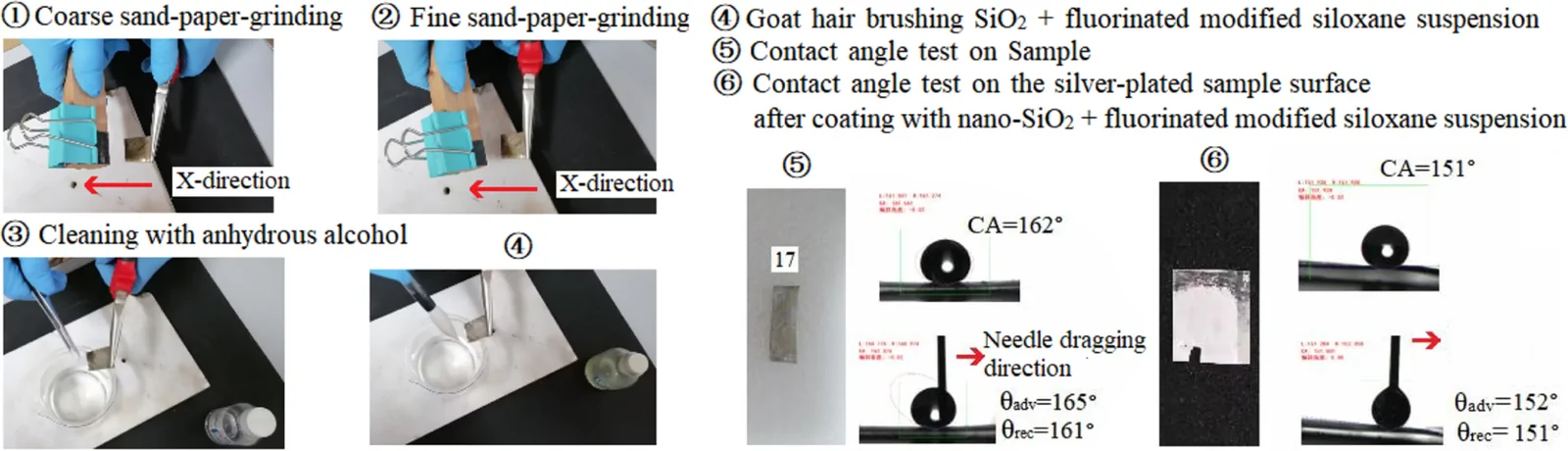

Study line for preparation of superhydrophobic surface based on Miao silver

**Specifications table**

**Table:** 

Subject area	Materials Science
More specific subject area	Materials Surface and Interface
**Name of your method**	Sand-grinding integrated with coatings
**Name and reference of original method**	H. Zhang, J. Li, J. Pan, Q. Mo, Structure-coatings two-step preparation for hydrophobic surface based on Miao silver together butterfly wing-simulated analysis, Materials Today Communications, 49 (2025) 114267.https://doi.org/10.1016/j.mtcomm.2025.114267
**Resource availability**	No other resource being reserved

## Background

Silver products, widely used in Miao ethnic clothing and vessels, are prone to corrosion and discoloration, seriously affecting the bright appearance. In electronic industry, the discoloration of silver also leads to increase of contact resistance of silver parts resulting in that the weldability and conductivity gets worse with it [1,2]. Therefore, how to prevent and slow down surface corrosion and discoloration of silver, and develop new anti-corrosion and anti-discoloration technology is a challenge to improve the application value of silver [3,4], especially for improvement of Miao silver jewelry and development of the local industry. The already studies indicate that corrosion and discoloration of the silver are affected by multi-factors as oxide (oxygen, nitrogen, chlorine), sulfide, residual salt, temperature, humidity, electromagnetic wave and so on (external factors) [3]; as well as properties of silver itself, coating performance, and process conditions (internal factors) [5].

The practical used Miao silver in ethnic clothing and vessels is mainly based on material of brass, white copper, or red copper instead of really pure silver. Freshly made Miao silver products are bright and white but easy **to be oxidized** and fade due to exposed to the air and no protection coatings. Therefore, how to reduce or prevent the oxidation and discoloration of silver ornaments requires further improvement in surface processing techniques, especially for the ability to collect and then repel the sweat [3,6,7]. Based on some success cases of anti-corrosion and discoloration by using the SHS [[8], [9], [10]], we also managed to use similar method to solve the problem of corrosion and discoloration for Miao silver to some extent.

Traditionally speaking, artificial SHS to simulate the organic superhydrophobic surfaces (lotus leaf, butterfly swing and water strider) is mainly based on two steps: firstly creating a surface with high roughness in micro/nano scale, then coating a hydrophobic materials with low surface energy [[11], [12], [13]]. We conducted this study to design an SHS with a micro-nano composite structure similar to the surface of water strider foot and butterfly wings by sandpaper-grinding the white copper and doping insoluble SiO_2_ nanoparticles into a fluorinated modified silane coating, and finally achieved an SHS with a certain degree of corrosion resistance. Thus both the sanding analogous to metal wire drawing (up-down mode) and the doping into coatings (down-up mode), obtaining an interface synergy effect (interaction of micro/nanostructure and chemical compositions), as a low cost and practical technique, are main experiment method different from other studies, and firstly applied to the Miao silver ornaments. Obviously, despite of our basic experiment method not breaking through the above traditional line but with own distinctive features in concrete technique style. Meanwhile, such method with general application property can be promoted to other requirements, i.e., silver contact materials for reduction of resistance, silver alloy solder, water repellence, seawater desalination, anti-biofouling, anti-fogging, antibacterial coatings and anti-icing of solid surfaces,oil-water separation and salt-tolerant evaporation[10,[14], [15], [16], [17], [18], [19]].

## Method details

This study mainly focuses on the preparation of hydrophobic surface and modeling to simulate the composite structure of butterfly wings, followed by property test and analysis of consistent between experiment and modeling. It mainly includes the following six aspects:

1. Theoretically, how to simulate micro/nano structure of butterfly wings by modeling based on the natural and artificial SHS, thus a semi-cylinder model was proposed.
2. Experimentally, how to prepare such a SHS similar to the butterfly wings’ composite structure; thus method of sand-grinding integrated with coatings was configured.
3. How to establish the relationship between microstructure and the roughness, solid fraction, contact angle, adhesion work and spreading coefficient based on the already formed composite wetting state.
4. Property test.
5. Data analysis (consistent between computation and experiment results).
6. Method validation.

Followed by explaining every aspect in detail from angle of methodology.

## Proposing a semi-cylinder model

Considering practical requirement of Miao silver or others, water-proof, anti-tarnishing/corrosion, and use of sand-grinding as an economic technology, we try to group them and create a superhydrophobic effect by designing a new method.

### Experimental basis for constructing the SHS

So far, because of the superhydrophobic phenomena from lotus leaf, taro leaf, water strider leg, butterfly wings in nature, strong curiosity drives people to study its secret. Accompanied with development of observation technique of microstructure, especially inventions of Scanning Electron Microscope (SEM) and Environmental Scanning Electron Microscope (ESEM), man have ability to observe the microstructure of materials. Finally, SEM and ESEM demonstrated the reason of “lotus effect” [20,21]. Taking a lotus leaf for example, as a representative of plant with hydrophobicity, on the surface of lotus leaf, there are innumerable frustum-shaped prisms in micrometer size (with an average diameter, spacing, and height of 8–10 µm, 10–12 µm, and 12–15 µm, respectively), and countless nanoscale protrusions (with an average height of 150 nm) being densely distributed on each prism and between them; meanwhile, the surface of these protrusions is covered with a hydrophobic waxy layer (Figs. 1(a)) [21]. Similarly, as a representative of animal with ability of water repellence, the surface of the water strider foot has countless bristle with an average length of 40–50 µm, and each bristle is covered with several nanoscale grooves (with length of >1 µm, and width of about 150 nm (Figs. 1(b) and (c))[20]. In addition, the surface of butterfly wings also has a scale-like micrometer structure (length of 100 µm, width of about 30 µm), on which the nanoscale cylindrical structure with length in micrometer, diameter about 1 µm; the column is also distributed with grooves and linear stripes between grooves with width 100 nm as the third level structure (Figs. 1(d) and (e)) [22]. Sum up, these organism structure enjoy a common characteristic: micro/nano double scale composite and structure fractal, the first-level structure with micrometer level ranging from 10 to 40 µm, and the second-level structure with nanometer level of about 100 nm. Although the above three microstructures differ greatly from one another in morphology, they all acquire e superior water repellence ability. Enlightened by such micro/nano structure, large number of artificial SHS’s progressively got to be fabricated. Just right, we are familiar with use of sand paper; butterfly wings’ micro-morphology ignites our creative line [22].

**Fig. 1:**
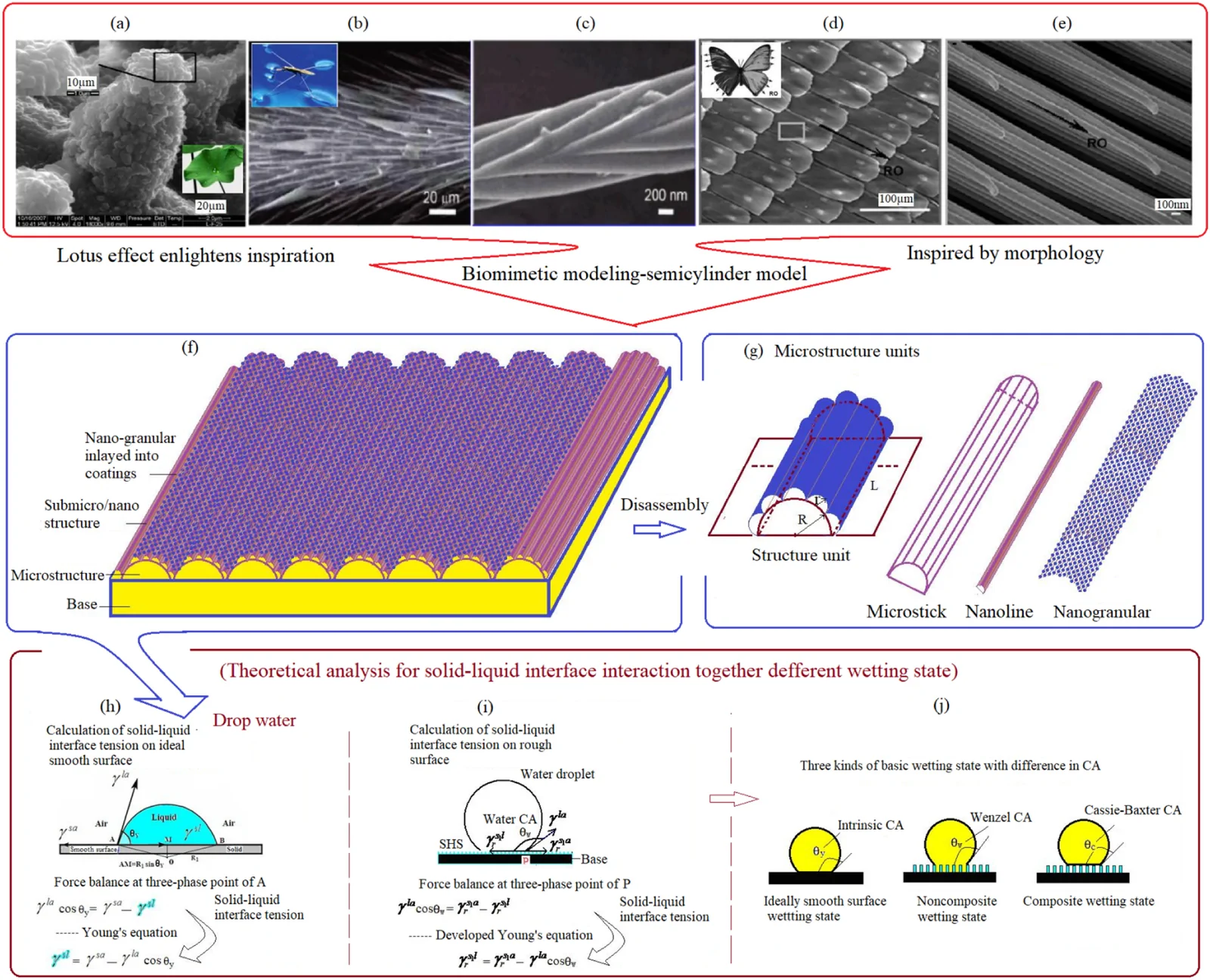
Semicylindrical model with micro-nano composite structure, simulating lotus leaf, water strider foot and butterfly wing. SEM image of the micro-nano structure. (a) Lotus leaf surface [21]; (b) Water strider leg surface [20]; (c) Nano-scale structure on the water strider leg surface [20]; (d) Micro-scale structure on the butterfly wing surface [22]; (e) SEM image of the nano-scale structure on the butterfly wing surface [22]; (f) Biomimetic modeling based on micro-nano structures of butterfly wings; (g) Structural components of the model, where the radius of the large and small semicircles in the structural units are R and r, respectively. Calculation of interfacial tension between solid-liquid interface; (h) an ideal smooth surface; (i) a rough surface; (j) three kind of wetting states with corresponding contact angle (CA) differences.

### Bionic modeling and line of preparation

Considering convenience of preparation, mainly referring the micro-morphology of butterfly wings and water striders' legs (Figs. 1(b)-(e)), a quasi-hemispherical composite structure (Figs. 1(f), (g)) has been theoretically designed based on the cylindrical grooves on butterfly wings, the steel hair bundles of water striders' legs. The hydrophobic coatings along with nano-granular in it (Fig. 1(g)) are similar to the wax substance with low surface free energy on the lotus leaf. The line connecting two intersection points of each nanoscale semicircle with microscale semicircle in cross-section is precisely the diameter of the nano-semicircle.

## Preparation of SiO_2_ powder + liquid fluorinated modified silane suspension

**Materials:**

A glass rod and glass mirror

**Chemicals and reagents:**

Fluorinated modified silane

SiO_2_ nanopowder (100 nm, surface tension of γ = 38.85 mN/m)

Fluorinated modified silane (surface tension of γ = 30∼35 mN/m)

**Equipment:**

Narrow-mouth bottle

Electronic scale

**Step-by-step protocol:**

1. Take 4 ml of fluorinated modified silane with a 100 ml narrow-mouth bottle.
2. Weigh 1 g of SiO_2_ by an electronic scale.
3. Pour **it** into the above narrow-mouth bottle.
4. Stir with a glass rod to mix SiO_2_ and fluorinated modified silane evenly to form a temporarily stable suspension (shorten for suspension next).

**Validation:**

Coating fluorinated modified silane twice on glass mirror, measured water CA of 105°, considered as the intrinsic CA of fluorinated modified silane.

## Preparing superhydrophobic surface using sand-paper grinding and nanoSiO_2_ doping

**Materials:**

A few pieces of white copper and Ag-5Cu (shorten for 95Ag) (as prepared), 100 mesh Al_2_O_3_ sandpaper, 1200 mesh SiC sandpaper, building blocks, goat hair (Fig. 2(a)-(d))

**Fig. 2:**
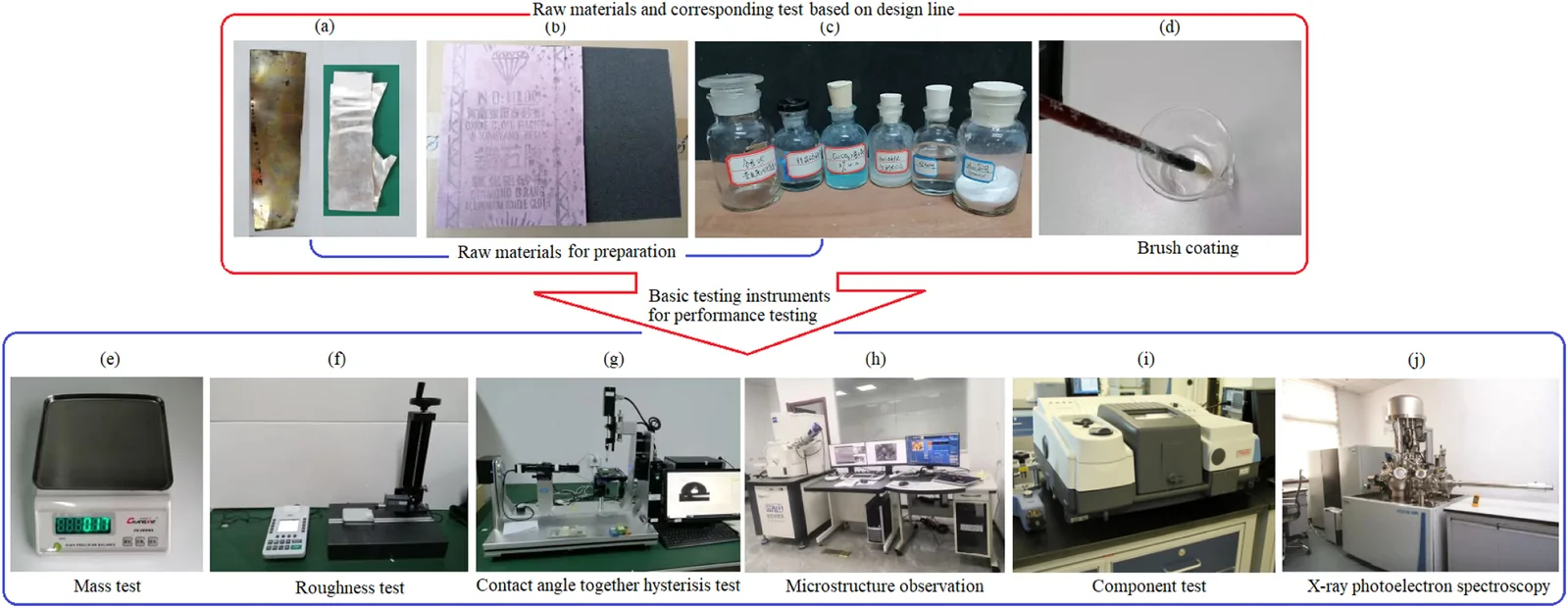
Experimental materials and testing instruments. (a) As prepared white copper (left) and Ag-5Cu (95Ag) sheet(right); (b) Sand paper; (c) Coating preparation material; (d) Goat hair dipped in coating; (e) Electronic scale; (f) Roughness measuring instrument; (g) Contact Angle measuring instrument; (h) Scanning electron microscope (SEM); (i) fourier transform infrared spectroscopy; (j) X-ray photoelectron spectroscopy of Thermo ESCALAB 250XI.

**Chemicals and reagents:**

Mixture of wax and rosin with ratio of 1:1

SiO_2_ nanopowder+fluorinated modified silane mixed suspension

3% salt solution and 5% copper sulphate solution for corrosion experiment

Colleseed oil and 20% salt solution for solid-air interface tension test based on Zisman method

**Equipment:**

Contact angle measurement instrument (SDC-350, origin: Shenzhen Daint Electronics Co., LTD. (Fig. 2(g)));

Roughness measurement instrument (Germany MarSurf M300C) (Fig. 2(f));

Scanning electron microscope (SEM: Zeiss sigma300) (Fig. 2(h));

High precision electronic balance (Dongguan Yixue Electronics: BA-2002 G) (Fig. 2(e));

Fourier transform infrared spectroscopy (FTIR, NICOLET6700, origin: Thermo company, USA) (Fig. 2(i));

X-ray photoelectron spectroscopy (XPS, Thermo ESCALAB 250XI, USA).

**Step-by-step protocol:**

Miao silver (copper sheet) selected as the substrate to simulate the two-tiered structures of butterfly wings and water striders' legs, as well as the nano-protrusions on lotus leaf surfaces by metal-drawing (or etching from up to down)[23]. For the first tier, a micrometer-scale structure was formed by coarsely grinding the copper sheet surface with 100-mesh coarse sandpaper to simulate the groove on butterfly wings and water striders’ legs; for the second tier, nano-scale structures were built on the mesoscale structure using 1200-mesh fine sandpaper to create a nano-semispherical structure on the mesoscale hemispherical structure. Additionally, immersing mutually insoluble nano-hydrophobic particles in liquid hydrophobic coatings can improve the hierarchical effects of micro-nano composite structures as shown in Fig. 1(g). Then, when applying suspension to the roughened surface of the components, a synergy effect for hydrophobicity appears by combining the silane, as a low-surface-energy material, with the aforementioned hierarchical structures [24]. Concrete operation step is as follows:

To grind out a rough structure in micron-level (Fig. 3(a), similar to hand drawing) on the base of white copper surface with coarse sandpaper (100 mesh Al_2_O_3_).

**Fig. 3:**
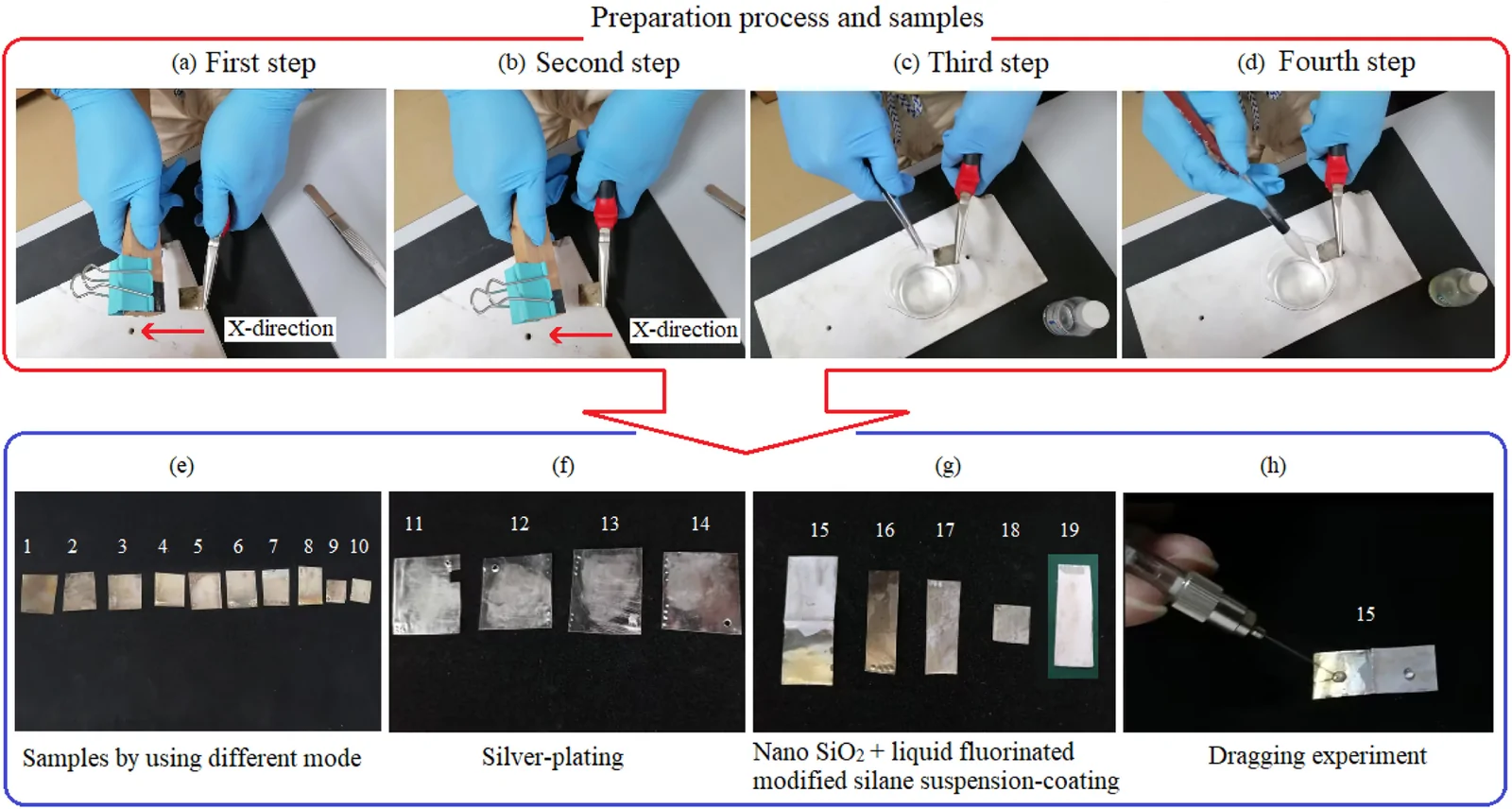
Preparation process, samples and water droplet drag experiment. (a) Coarse grinding**.** (b) Fine grinding**.** (c) Anhydrous alcohol clearing**.** (d) Goat hair brushing**.** (e) Samples**.** (f) Silver-coated samples**.** (g) Samples coated with nano SiO_2_ + liquid fluorinated modified silane suspension**.** (h) Drag the water droplets on both halves of the sample 15 with a syringe needle.

Take 5 samples of different white copper plates, number them, and grind it with 100 mesh Al_2_O_3_ sandpaper for 10 times, 20 times, 30 times, 40 times, respectively, moreover applying a force of from 15 to 20 Newton each time regardless of grinding direction or sandpaper, to form microstructure grooves with certain orientation (assume to be in the direction of X-axis) so as to imitate the microscale steel hair of water strider's foot. In terms of the effects of experiment, coarse/fine sanding 20 times each in the same direction can achieve a better result of superhydrophobicity. As experience, the follow-up preparation will adopt the method used for sample 8 temporary.

Fine sandpaper (1200 mesh SiC) was used to grind out nano-scale structure on the microstructure (Fig. 3(b)).

The SiC sandpaper was used to grind the white copper sheet in the same direction as above assumed 10 times, 20 times or 30 times, respectively, so as to form finer microscale stripes on the ridges between the microscale grooves, imitating the nanoscale stripes on the bristle and forming a two-level composite microstructure.

Clean the sample surface with anhydrous alcohol and dry it (Fig. 3(c)).

Use goat hair to dip in the above suspension and brush it on the sample, applying it 1–3 times for different samples. After natural drying for 5 h, a hydrophobic surface with micro-nano composite structure and low surface energy coating similar to these of lotus leaves and water strider legs was formed (Fig. 3(d)).

The obtained samples were shown in Fig. 3(e)-(g). The samples numbered 1–10 correspond to the treatment instructions in Table 1; the methods used for roughening numbered 11–14 correspond to those of samples 8, 7, 6, and 2, followed by silver-plating and coating. The results show that tablet 11 has the highest CA after silver plating. Samples 15, 17, 18, and 19 (used for SEM testing, and comparison and verification) have the same roughening treatment as sample 8; while sample 16, which is the original white copper tablet, serves as a reference. Fig. 3(h) shows a comparison experiment for the water droplet to be dragged by syringe tip on sample 15 with half of front surface roughened together coated by suspension and the other half keep as-prepared (shorten for drag experiment later).

**Table 1:** Structure and performance test data of hydrophobic surface prepared with Miao silver as the base.

Sample number	1	2	3	4	5	6	7	8	9	10	19
Sample treatment method corresponding to number	1. Untreated white copper sheet. 2. Coarse/fine sand papers sanded successively at 90° in cross pattern both x and y axis for 10 times each without applying a coating. 3. Done for 10 times **each**, coating 2 times. 4. Done for 20 times **each**, coating 2 times. 5. Done for 30 times **each**, coating 2 times. 6. Coarse/fine sandpapers sanded successively in same direction for 10 times each, coating 2 times. 7. Same to 6, respective for 20 and 10 times, coating 2 times. 8. Same to 6 for 20 times each, coating 2 times. 9. Same to 6, respective for 30 and 10 times, coating 2 times. 10. Same to 6, respective for 40 and 10 times, coating 2 times. 19. Same to 6, respective for 20 and 20 times, coating 2 times.										
R_a_/µm*	0.23	0.81	1.22	0.87	1.32	0.37	0.54	1.87	0.66	0.72	1.50
H	2.52	2.65	2.76	2.67	2.78	2.54	2.59	2.98	2.67	2.63	2.83
CA_f_(°)	69	64	106	107	107	112	123	131	108	113	110
CA_sio2_(°)	–	–	–	–	–	–	155	162	–	–	154
CA_adv_/CA_rec_(°)	–	–	–	–	–	–	–	165/161	–	–	152/151
CA_20_(°)	–	–	–	–	–	–	152	154	–	–	151
CA_NaCl_(°)	–	–	–	–	–	–	–	138	–	–	152
w_a_(mJ)	100.67	106.60	53.69	52.44	52.44	46.35	6.94	3.63	51.22	45.16	7.37
γ_r_^sa^(mN)	71.30	54.85	364.29	386.80	386.80	443.49	1022.52	1080.51	399.70	457.23	1014.35
γ_r_^sl^(mN)	44.70	22.40	384.70	408.50	408.50	471.30	1089.70	1151.00	432.50	486.20	1079.40
S_l/s_(mJ)	−47.55	−41.62	−95.54	−95.78	−95.78	−101.88	−141.28	−144.60	−97.0	−103.07	−138.23

* Note:.

(1) Description of property quantities: *R_a_*-arithmetic mean roughness; CA_f_-water CA after coating fluorinated modified silane; CA_sio2_-water CA after coating suspension (mixing fluorinated modified silane with nano SiO_2_ powder as mentioned above); CA_adv_/CA_rec_ advanced/receding CA; CA_20_-water CA after 20 days; CA_NaCl_-water CA after 20 days of brine corrosion; *w_a_*-adhesion work per unit of solid-liquid area; *S_l/s_*-spreading coefficient per unit area; *γ_sl_*-real solid-liquid-interface tension (or surface energy) per unit area; (*w_a_, S_l/s_* and *γ _r_^sl^*, indirect test of sample 8 based on 162°). Coating fluorinated modified silane twice on glass mirror, measured water CA of 105°, considered as the intrinsic CA of fluorinated modified silane.

(2) Transformation of linear roughness of R_a_ into area roughness (or 3-D roughness ratio of *H*), refer to Fig. 4 and Eq. (2) and Eq. (5).

(3) Solid-air interface tension (*γ_r_^sa^*) and solid-liquid interface tension (*γ_r_^sl^*) were indirectly calculated by using software carried by CA measurement instrument (SDC-350) based on OWRK method; meanwhile use liquid 1 of water and liquid 2 of glycol (with low surface energy) and make glycol CA approximately equal zero when contact with samples 1–10 (**mental** substrate as hydrophilic materials) due to low surface energy to avoid negative solid-air interface tension. Therefore both values of γ_r_^sa^ and *γ_r_^sl^* are the computation results at specific conditions.

The following steps only belong to property test instead of preparation. The detailed operation see “5. property test” later.

Roughness test

After samples (numbered as 1–19 shown in Fig. 3(e)-(g)) have been prepared, corresponding one-dimension roughness can be measured using roughness measurement instrument, including naked surface and coated surface.

Aging and anti-corrosion test (Fig. 5).

Contact angle together hysteresis test

Direct property test mainly includes water CA, CAH (by means of dragging using needle of syringer instead of sessile drop method) obtained by SDC-350. However, adhesion work (*w_a_*), solid-air/solid-liquid interface tension (γ_r_^sa^, γ_r_^sl^) and spreading coefficient (S_l/s_) belong to compound quantities, **only** by indirectly calculating and computing software attached by contact angle measurement instrument (SDC-350) itself. Test data and visualization of theoretical relationship between different parameters and microstructure are as Table 1 and Fig. 6 show [25].

(1) Observation of infrared spectrum by fourier transform infrared spectroscopy (Fig. 7(a)-(c)).
(2) Observation of X-ray photoelectron spectroscopy (XPS) (Fig. 8(a)-(b)).
(3) Observation of microstructure by scanning electron microscope (Fig. 7(d)-(f) and Fig. 8(c)-(d)).

**Validation:**

After test and observation, the related data were recorded in Table 1. The key result of sample 8 is as: static apparent CA of 162°, advancing/receding CA of 165°/161°respectively. The qualitative and intuitive experiment is as Fig. 3(h) shows (see “video of needle dragging water droplet” as supplementary materials).

**Troubleshooting:**

Our study has come to basic destination for achieving the SHS, but the ability to anti-corrosion still awaiting for improved (Fig. 5(e), (f), (c), (g)).

## Establish analytical relationship between microstructure and the property parameters based on the already formed composite wetting state

To our knowledge, it is micro/nano-structure that creates the superhydrophobic effect. Therefore, series of property parameters, i.e., roughness, solid fraction, contact angle, adhesion work and spreading coefficient, must be associated with microstructure parameters, such as radius of semi-cylinder model, coatings chemical compositions (approximately represented by intrinsic CA on the ideal smooth surface).

**(1) Roughness (or harshness→ H (or ϒ))**

For the rough surface by coarse/fine sandpaper grinding in the same direction, a similar semi-cylindrical model as shown in Fig. 1(f) can be created to simulate the microstructure of butterfly wings (Fig. 1(e)). According to the structure unit in Fig. 1(g), the theoretical surface roughness can be calculated as

(1) $$H=\frac{\pi RL}{LR}\times \frac{L\pi r\times \left(\frac{\pi }{2\arcsin\frac{r}{R}}\right)}{\pi RL}=\frac{\pi r}{R}\left(\frac{\pi }{2\arcsin\frac{r}{R}}\right)\underset{\to }{Givenr<<R}\approx \frac{\pi RL}{LR}\times \frac{L\pi r\times \left(\frac{\pi R}{2r}\right)}{\pi RL}=\frac{\pi^{2}}{2}\approx 4.93$$

where the theoretical approximation is a constant, much higher than the critical roughness for the transition from non-composite to composite wetting state, favoring the formation of composite wetting state [26,27].

About this transition, it is no doubt there has been practical significance for surface to waterproof, also the purpose of this study. Actually, its realization suffer from multi-factors, i.e., morphology, surface energy, temperature and humidity, vibration of water droplet and purity, especially roughness [26]. Therefore, corresponding critical condition for transition from non-composite to composite wetting state is not absolutely. Only the outside environment and materials of solid-liquid interface given, corresponding critical condition is still certain, i.e., under certain outside environment and materials, such critical condition depend mainly on roughness of solid surface, and critical roughness change with hydrophobicity of materials, more hydrophobic, lower critical roughness. We once obtained 1.8 or so of value based on two-step microstructure and 120° of intrinsic CA based on equal adhesion work by linking non-composite with composite wetting state; earlier value of 1.75 in 1964 from Rulon E. Johnson Jr. and Robert H. Dettre’s research based on equal apparent CA using Wenzel equation and Cassie-Baxter equation [26,28]. Higher roughness can inhibit the formation of non-composite wetting state. Therefore this study specially highlights the roughness of surface by designing composite micro/nano structure, satisfying the condition of transition from non-composite to composite wetting state. Significantly, the roughness of surface model from computation is 4.93, being far higher than the critical value, no doubt that formation of composite wetting state.

The computation results largely satisfy with the actual hydrophobic state of the lotus leaf and water stride’s leg. Furthermore, we found that the radius (r) of nano-semi-cylinder has key effect to the surface property once the composite wetting state is formed. However, both radius directly and comparably affect the roughness and solid fraction respectively (Fig. 6(a_1_) and 6(a_2_)). More detailed discussion may refer to our previous study [26].

Especially note that both radius of *R* and *r* only used for configuration of microstructure respective in micro-size and nano-size, its change in size will not influence morphology of coatings, namely, the morphology keeps unchanged, also the roughness of 4.93 at least from theoretically modeling. Certainly, there exist differences in the morphology experimentally due to sand-grinding differences in direction and force.

For real rough surface of 95Ag sheet, because of not to directly test 3D roughness of surface, followed by an important question of how to transform one-dimensional (1D) line roughness of *R_a_* (test result of this study by using Germany MarSurf M300C) into area roughness (3D), if given average roughness of *R_a_*, based on Eq. (2), we can obtained the 3D roughness, at least approximately, despite of the rough surface not being absolutely isotropy [29,30].

(2) $$H\approx r_{1}^{2}$$

where *r_1_* stands for 1-dimension line roughness ratio. Among it, key to it is how to find *r_1_.* Based on our semicylindrical model and the average roughness *Ra*, an ideally periodic arc-shaped microstructure (to simulate coarse/fine sand-grinding surface) and single-layer spherical nanostructure (to imitate nano-SiO_2_ coatings directly contact with microstructure) was designed to find micro-line roughness ratio of r_1m_ (Fig. 4(a)) and nano-line roughness ration of r_1n_ (Fig. 4(b)) as Eq. (3) and (4) show.

(3) $$r_{1m}=\frac{\left(\arcsin\left(\frac{\sqrt{R_{1}^{2}-{\left(R_{1}-R_{a}\right)}^{2}}}{R_{1}}\right)\right)R_{1}}{\sqrt{R_{1}^{2}-{\left(R_{1}-R_{a}\right)}^{2}}}$$

where *R_a_* = 1.8 μm stands for *the average line roughness from the test results of* Germany MarSurf M300C, *R_1_* =15.3μm for the average diameter of sand grain of 1200-mesh fine sand-paper. For nano-SiO_2_ coatings, suppose it as single layer structure with grain,

(4) $$r_{1n}=\frac{\pi R_{2}}{2R_{2}}=\frac{\pi }{2}\approx 1.57$$

where *R_2_* stands for average radius of nanogranular of SiO_2_, i.e., 50 nm. It indicates that 1-dimension line roughness ratio r_1n_ is irrelevant to diameter of grain radius (*R_2_*) due to morphology constant. Integrating Eq. (3) and Eq. (4), total line roughness ratio of rough surface with micro/nanostructure may be represented as Eq. (5),

(5) $$r_{1}=r_{1m}\times r_{1n}=\frac{\left(\arcsin\left(\frac{\sqrt{R_{1}^{2}-{\left(R_{1}-R_{a}\right)}^{2}}}{R_{1}}\right)\right)R_{1}}{\sqrt{R_{1}^{2}-{\left(R_{1}-R_{a}\right)}^{2}}}\times \frac{\pi }{2}$$

**Fig. 4:**
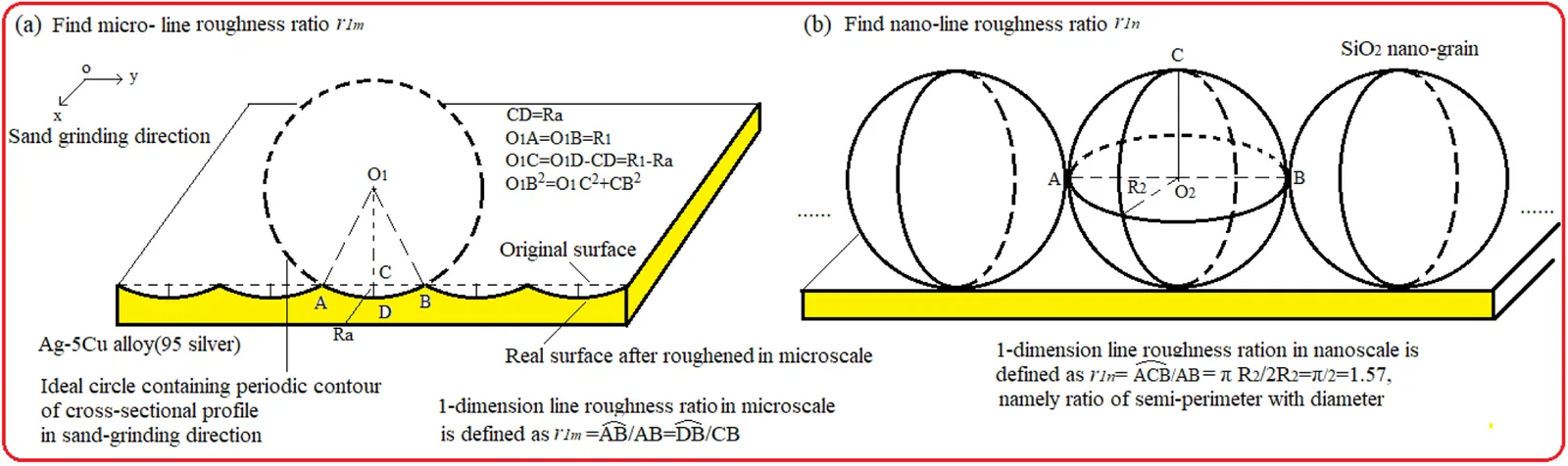
Find 1-dimension line roughness ratio *r_1_* from micro/nano scale respectively.

It shows that total line roughness ratio of *r_1_* is only related with diameter of micro-sand grain (*R_1_*) and test value of *R_a_* . According to test results, *R_a_* = 1.8 μm, *R_1_* = 7.6 μm (average grain radius of 1200 mesh sandpaper), substitute it into Eq. (5), obtain *r_1_* = 1.724, thus *H* = *r_1_^2^* *=* *2.93*, which is obvious larger than the critical roughness for transformation from non-composite to composite wetting state for hydrophobic materials[26,28]*.* Although there exist apparent difference compared with the results of Eq. (1), that the obtained roughness exceeds critical value agrees with each other. Then substituting Eq. (5) into Eq. (2), the values of area roughness all are larger than 2 (Table 1), exceeding the critical value, which demonstrates the model with characteristic to form composite wetting state.

**(2) Determination of CA**

For the ideal smooth surface, Yang Eq. (6) is used to describe the WS of solid-liquid interface (Fig. 1(h), (j)),

(6) $$\cos\theta_{Y}=\frac{\gamma^{{}_{sa}}-\gamma^{{}_{sl}}}{\gamma^{{}_{la}}}$$

For rough surface, the non-composite state is described by Wenzel Eq. (7) (Fig. 1(j)),

(7) $$cos\theta_{w}=Hcos\theta_{y}$$

where *H* is the surface roughness of the solid surface, as in Eq. (1).

The composite state is described by the Cassie-Baxter Eq. (8) (Fig. 1(j)),

(8) $$\cos\theta_{\text{ca}}=f_{s}\left(\cos\theta_{s}+1\right)-1$$

where *θ_s_* is the intrinsic CA of the corresponding solid part, and ƒ_s_ is the fraction of solid parts (Eq. (13)). Naturally, the CA together hysteresis are affected by multiple factors, of which physical mechanism is complicated, including surface chemical compositions, microstructure and morphology, even environmental conditions [12,31].

**(3) Quantitative contact angle hysterisis (CAH)**

According to the existing research of our group, CAH can be quantified and expressed as Eq. (9) for non-composite wetting state,

(9) $$\begin{array}{c} CAH=\theta_{a}-\theta_{r}=2\left[arctg\left(c_{a}tg\frac{\theta_{w}}{2}\right)-arctg\left(c_{r}tg\frac{\theta_{w}}{2}\right)\right]\\ =2\left[arctg\left(1.15\varphi \left(\theta_{y}\right)\right)-arctg\left(0.89\varphi \left(\theta_{y}\right)\right)\right] \end{array}$$

where

(10) $$\phi \left(\theta_{y}\right)=tg\frac{\theta_{w}}{2}=\sqrt{\frac{1-H\cos\theta_{y}}{1+H\cos\theta_{y}}}$$

where *θ_y_* the intrinsic CA of a smooth surface [32]. For composite wetting state, it can be expressed as [32]

(11) $$\begin{array}{c} CAH=\theta_{a}-\theta_{r}=2\left[arctg\left(c_{a}tg\frac{\theta_{ca}}{2}\right)-arctg\left(c_{r}tg\frac{\theta_{ca}}{2}\right)\right]\\ =2\left[arctg\left(1.15\xi \right)-arctg\left(0.89\xi \right)\right] \end{array}$$

where

(12) $$\xi =tg\frac{\theta_{ca}}{2}=\sqrt{\frac{2-\left(1+\cos\theta_{s}\right)f_{s}}{\left(1+\cos\theta_{s}\right)f_{s}}}$$

where *θ_s_* is the intrinsic CA of a smooth surface. For composite wetting state, the solid fraction (f_s_) can be expressed as

(13) $$f_{s}=\frac{2\pi \left(1-\frac{\theta_{s}}{180}\right)}{2\pi \left(1-\frac{\theta_{s}}{180}\right)+\left(2R-2r\sin\theta_{s}\right)}\overset{while\theta_{s}=105^{\circ }}{\to }\approx \frac{0.8\pi }{0.8\pi +\left(2R-1.94r\right)}$$

where *θ_s_* is the intrinsic CA, corresponding to the solid surface. For the semi-cylindrical model, it can be calculated by surface area integral.

**(4) Adhesion work on the unit solid-liquid interface (shorten for adhesion work)**

The adhesion work on the solid-liquid interface reflects the interaction between solid and liquid molecules, expressed by Yang-dupre Eq. (14),

(14) $$w_{a}=\left(\gamma_{r}^{sa}+\gamma^{la}-\gamma_{r}^{sl}\right)=\gamma^{la}\left(1+\cos\theta_{w}\right)$$

**(5) Spread coefficient**

The spreading coefficient is used to quantify the level of wetting of a liquid on a solid surface, expressed as Eq. (15)

(15) $$S_{l/s}=\gamma_{r}^{sa}-\left(\gamma^{la}+\gamma_{r}^{sl}\right)=\gamma^{la}\left(\cos\theta_{w}-1\right)$$

It indicates that larger CA, lower *S_l/s_*(<0), and weaker wetting.

**(6) Solid-liquid interfacial tension**

Water droplets form an interface with the coatings, of which the interfacial tension reflects whether a phase transition is evident. The greater the interfacial tension, the more pronounced the solid-liquid-phase separation, indicating weaker interactions between them, thus reducing adhesion and making it easier for the liquid droplet to roll off the solid surface. For an ideal smooth surface (Fig. 1(h)), Yang's equation provides the relationship between this force and the intrinsic CA,

(16) $$\begin{array}{c} \gamma^{{}_{sa}}=\gamma^{{}_{sl}}+\gamma^{{}_{la}}\cos\theta_{Y}\Rightarrow \gamma^{{}_{sl}}=\gamma^{{}_{sa}}-\gamma^{{}_{la}}\cos\theta_{Y}\\ \text{or}\gamma^{{}_{sl}}-\gamma^{{}_{sa}}=-\gamma^{{}_{la}}\cos\theta_{Y} \end{array}$$

where *θ_y_* is the intrinsic CA of water droplets on a smooth surface. For actual rough surfaces (Fig. 1(h)), the modified Yang equation can also give the relationship between the interfacial interaction force and the apparent CA,

(17) $$\begin{array}{c} {\gamma_{r}}^{{}_{sa}}={\gamma_{r}}^{{}_{sl}}+\gamma^{{}_{la}}\cos\theta_{w}\Rightarrow {\gamma_{r}}^{{}_{sl}}={\gamma_{r}}^{{}_{sa}}-\gamma^{{}_{la}}\cos\theta_{w}\\ or{\gamma_{r}}^{{}_{sl}}-{\gamma_{r}}^{{}_{sa}}=-\gamma^{{}_{la}}\cos\theta_{w} \end{array}$$

where *θ_w_* is the apparent CA of water droplet on rough surface. Theoretically, when the rough solid surface is given, larger apparent CA (*θ_w_*) implies stronger interfacial tension between solid and liquid, thus reducing adhesion, and improving the rolling effect of droplet [27].

## Property test

### Contact angle test

Direct property test mainly includes water CA, CAH (by means of dragging using needle of syringer instead of sessile drop method) obtained by SDC-350. However, the roughness (aim at area), solid fraction, adhesion work and spreading coefficient belong to compound quantities, only by indirectly calculating and computing software attached by SDC-350 itself. Test data are as Table 1 shows [25].

### Syringe dragging test

Take sample 15 **sheet** (2 cm × 4 cm), roughen one-half using the No.8 method, and then brush it with suspension three times; the other half remains unchanged. Then drop 10 µl of water on each half, insert the needle tip to drag the water droplets (Fig. 3(h)). Capture a specific dragging state, and measure the CA at the front and back edges simultaneously, which are approximately advancing/receding CA, of which **their** difference is the CA hysteresis (shorten for CAH next). Therefore, this study does not use Sessile Drop (injection/absorption) method to measure the advancing/receding CA.

### Aging and anti-corrosion test

Set normal temperature and indoor environment, place sample 8 in such condition for 30 days, the CA and CAH almost do not suffer from changing, indicating that the environment under normal outdoor air conditions will not have a significant impact on the coatings, and the coatings are durable to aging (Fig. 5(a), (b), (e), (f)).

**Fig. 5:**
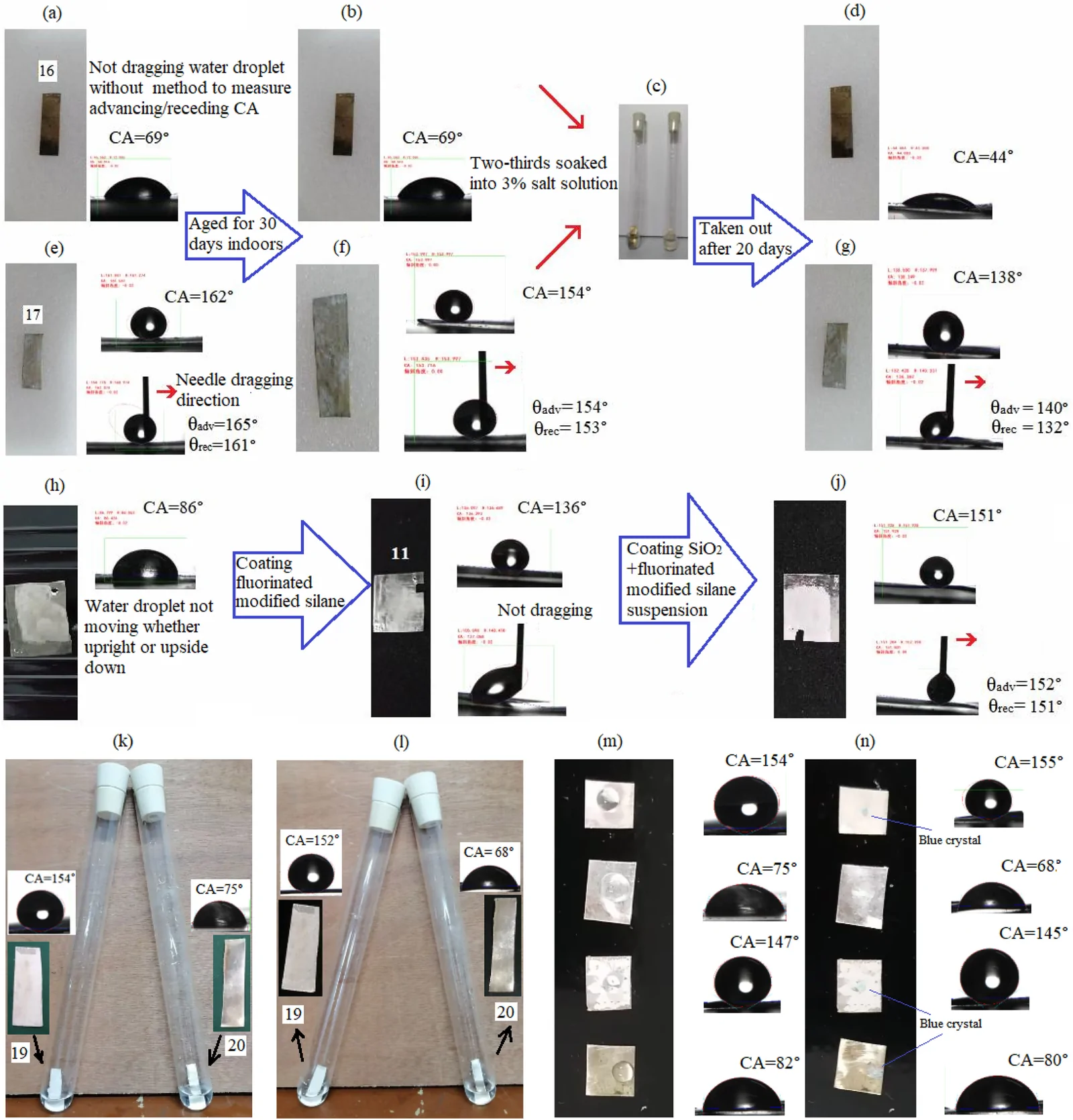
Aging and salt resistance test, water contact angle together hysteresis test. (a) Original white copper sheet of sample 16; (b) Sample 16 aged for 30 days; (c) Samples 16 and 17 soaked in a 3% salt solution for 20 days, with 2/3 immersed; (d) Sample 16 removed from the salt water; (e) Sample 17. (f) Sample 17 aged for 20 days; (g) Sample 17 removed from the salt water; (h) Silver-coated CA test on sample 11; (i) Fluorinated modified siloxane contact angle test on Sample 11; (j) Contact angle test on Sample 11 after coating with nano-SiO_2_ + fluorinated modified siloxane suspension; (k) Sample 19 and as-prepared 95Ag sheet soaked in a 3% salt solution for 20 days, with 1/2 immersed; (l) Sample 19 and as-prepared 95Ag sheet respectively taken out from the salt water 20 days later; (m) copper sulfate drop test respective for sample 19, as prepared 95Ag, sample 15, as prepared white copper from up to down, copper sulfate drop on surface at initial state; (n) water in copper sulfate liquid drop evaporated up results in copper sulfate crystal left (see blue sign) after 19 h.

Whether having anti-corrosion ability or not, referring to Chinese standards GB/1763–89 (test Method for Chemical Resistant Properties of Paint Coatings), firstly use mixture of wax and rosin with ratio of 1:1 to edge-banding, then soak 2/3 of sample 8 (white copper substrate+coatings) and 1/2 of sample 19(95 Ag substrate+coatings) into 3% salt solution for 20 days. The tested CAs fall down to 138° from 161° (Fig. 5(e)-(g)), to 152° from 154° (Fig. 5(k)-(l)), respectively. The hydrophobic performance decrease greatly for **white substrate**, but lightly slow for 95Ag substrate owe to weak chemical activity of Ag. Both surface coatings show signs of swelling, suggesting that the corrosion resistance to salt solution is poor and temporarily can not meet the practical requirements, needs to be further optimized.

Further copper sulfate drop test approves above results. After 19 h contact with copper sulphate, 4 samples: sample 19, as prepared 95Ag, sample 15, as prepared white copper do not show obvious corrosion signs only leaving blue crystal due to all water being evaporated. After cleaning, retest water CA with 155°, 68°, 145°, 80°, only lightly decrease in CA, demonstrating coatings and substrates with certain anti-corrosion capabilities (Fig. 5(m) and 5(n)), at least for short time contact, because the chemical activity of silver is less than that of copper.

On account of weak anti-corrosion ability, impedance between coatings and conductive substrate (white copper and 95Ag) also shows minor change, i.e. before immersing tends to ∞, later shows some limit values, even zero value because of coatings possibly not being uniform in thick thus corroded in some point, leading to short circuit.

## Data analysis

Select 10 **pieces of** samples for direct or indirect testing, test content includes roughness, solid fraction, CA together hysterisis (Table 1), suface/intersurface tension, adhesion work and spread coefficient, SEM observation and Infrared spectrum (Fig. 6).

**Fig. 6:**
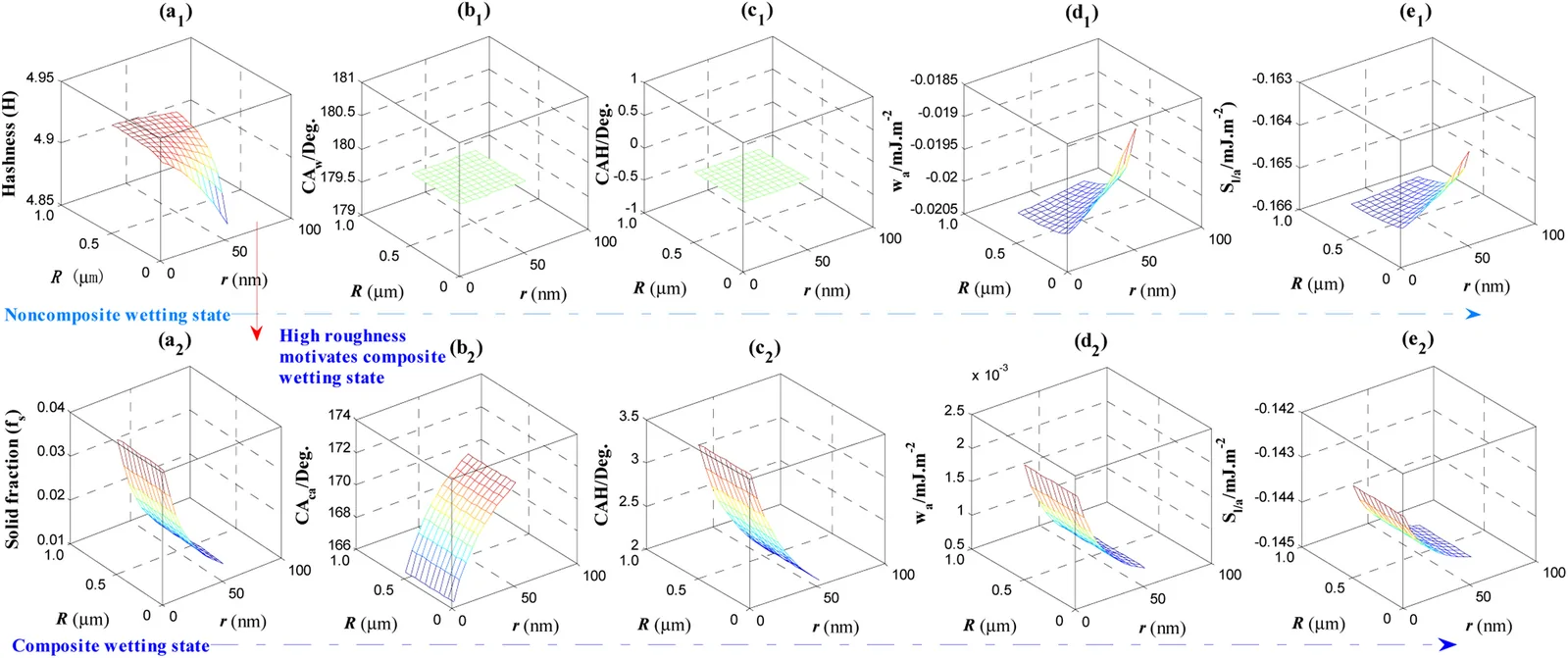
Variations of the surface property with radius of micro/nano semi-cylinder (*R* and *r*) in composite wetting state (CWS). (a) The solid fraction (f_s_); (b) Cassie’s contact angle (CA_ca_); (c) Contact angle hysteresis (CAH); (d) Normalized adhesion work of a water droplet; (e) Spreading coefficient of water.

### Roughness together solid fraction determines theoretical and experiment results

According to Eqs. (1) and (13), the area roughness and solid fraction in possible composite wetting state are as Fig. 6(a_1_), (a_2_) indicate. Computation results of the roughness demonstrate its increase with radius of micro semi-cylinder, and inversely with radius of nano semi-cylinder. When higher area roughness (larger than 1.8) satisfy condition for forming composite wetting state, solid-liquid interface possibly enter into composite wetting state instead of non-composite wetting state, thus gaining large CA, less CAH and adhesion work together spreading coefficient (Fig. 6(b_2_)-(e_2_)). Correspondingly, experiment test largely accords with computation, i.e., with increase in arithmetic mean roughness of the substrate from sample 1 to 8, after coating fluorinated modified silane, water CA also increase with it from 64° to 131°. If coating suspension (obtained by doping SiO_2_ into fluorinated modified silane) on the substrate of sample 8, water CA continues to increase up to 162°, advancing/receding CA respectively up to 165°/161°, adhesion work of 3.627 mJ (Table 1). Yet the computation results, based on the composite wetting state, are that water CA change from 160° to 170°, CAH of 3° or so, adhesion work of 1.5 mJ or so (Fig. 6(d_2_)). Simulation and experiment results largely agree with each other, which prove validity of the study line.

### Differences in chemical compositions and micro-morphology result in different surface property

Despite that the microstructure of surface is the key to the superhydrophobic phenomena, chemical compositions (infrared spectrum in Fig. 7(a)-(c)) and micromorphology (SEM in Fig. 7(d)-(f)) also cannot be ignored; different affiliated absorption peak and SEM structure reflect the changes in surface chemical compositions and micromorphology, respectively. Compared with the as-prepared white copper (infrared spectrum showing main absorption peaks at 575.84 cm^−1^, 1044.31 cm^−1^, affiliated absorption peak at 780.97 cm^−1^, 1535.13cm^−1^, 1623.39cm^−1^, 1678.16cm^−1^, Fig. 7(a)), the fluorinated modified silane (infrared spectrum showing main absorption peak at 800.46 cm^−1^, 1727.25 cm^−1^, affiliated absorption peak at 566.78 cm^−1^, 742.75cm^−1^, 954.73cm^−1^, 1678.16cm^−1^, 2959.65 cm^−1^, Fig. 7(b)), nano SiO_2_ particles (infrared spectrum showing absorption peak at 802.49 cm^−1^, 1069.47 cm^−1^, affiliated absorption peak at 950.82 cm^−1^, Fig. 7(c)), as low surface energy materials, concurrently play the pivotal role in improving roughness and reducing surface energy of the as-prepared white copper in the composite structure. The SEM of sample 8 (Fig. 8(c)) shows the distribution of nano SiO_2_ particles on the cylindrical groove (Fig. 8(b)). Similarly, from the as-prepared white copper (69°)→ roughened (64°) →coating silver (86°) →coating nanofluorinated modified silane (136°) →coating silica suspension (162°), the CA increases significantly and the CAH decreases sharply. As surface arithmetic mean roughness changes (Table 1) and different infrared spectrum indicates composition change (Fig. 7(a)-(c)), both surface roughening and low surface energy coatings synchronously affect on the waterproofing of the silver-coated film (Fig. 5(i)-(j)).

**Fig. 7:**
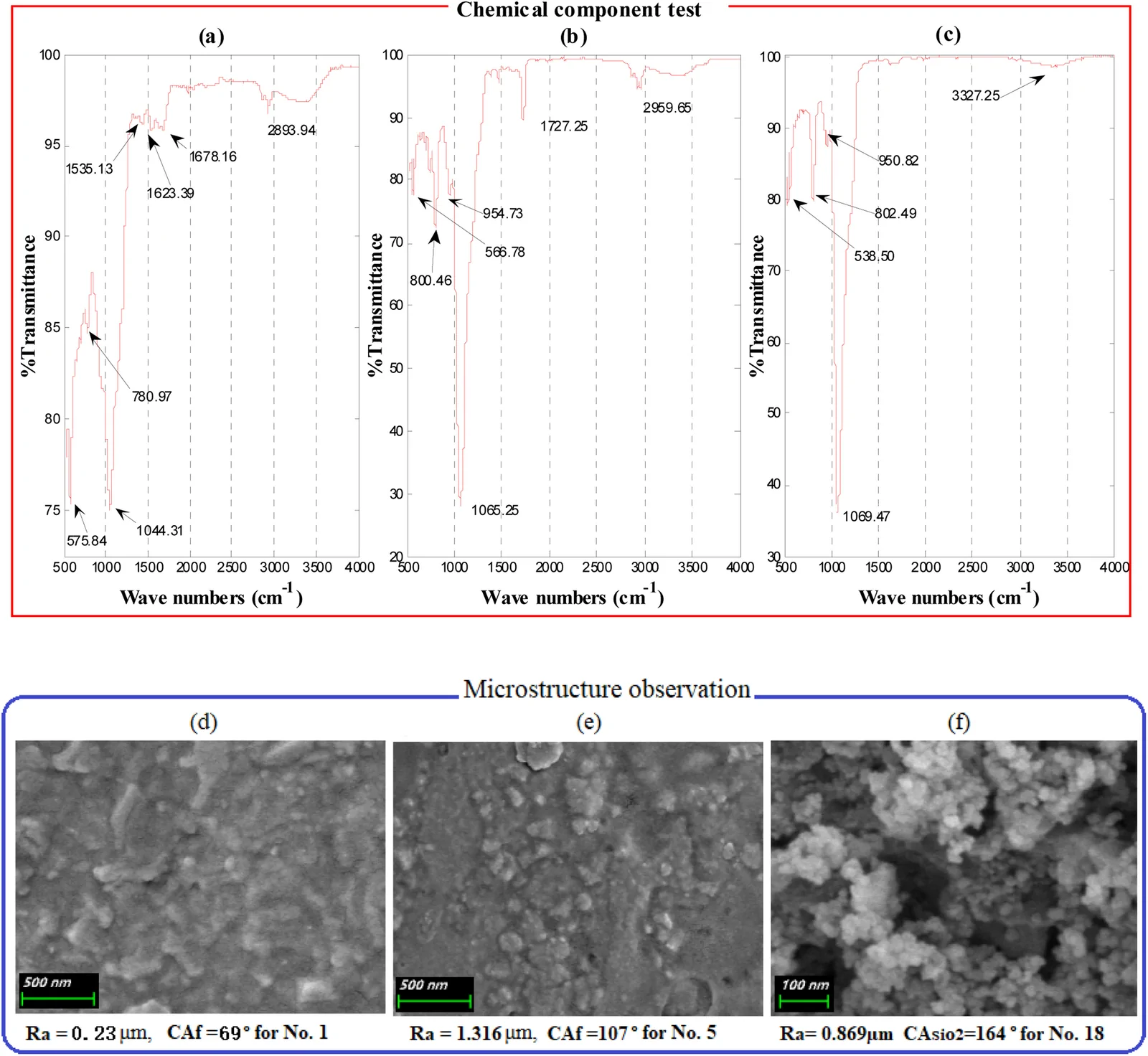
Infrared spectrum of (a) sample 1 of as-prepared white copper sheet; (b) sample 7 of the roughened white copper sheet followed by silver-coated, finally coated with fluorinated modified silane; (c) sample 8 of the roughened white copper sheet followed by coating with suspension of SiO_2_ powder + liquid fluorinated modified silane; SEM image of (d) as-prepared sample 1; (e) sample 5; (f) sample 18 (microstructure and coatings corresponding to sample 8).

**Fig. 8:**
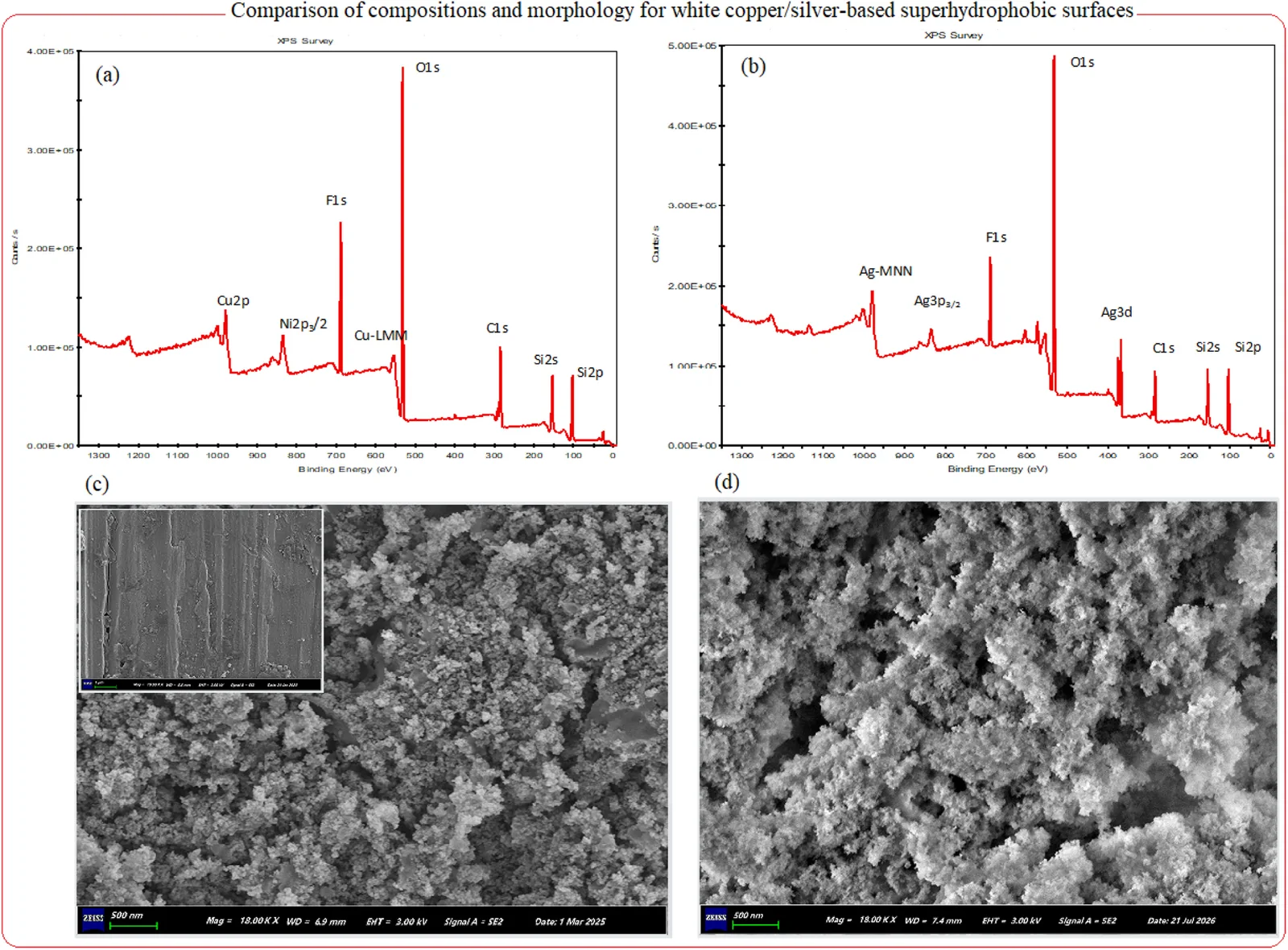
X-ray photoelectron spectrum ((a) and (b)) and images of scanning electron microscope ((c) and (d); inset of (c) for microstructure of substrate in micro-scale, as first step of composite structure, green scale bar in it is 1μm) respective for white copper-based ((a) and (c)) and 95Ag-based ((b) and (d)) superhydrophobic surface.

As supplement, we selected 95Ag for further verification (sample 19 in Table 1), the method for roughening surface is same to sample 8, and series of property parameters are also presented in Table 1, of which change in the CA from 75°(as-prepared silver), 72° (being roughened), 135° (coated nanofluorinated modified silane), to 154°(coated silica suspension), indicating improvement of the hydrophobicity step by step. Meanwhile, X-ray photoelectron spectroscopy (XPS) also test the existence of fluorinated modified silane and SiO_2_ (Fig. 8), demonstrating the positive effect of hydrophobic materials and microstructure in superhydrophobicity. Although the base materials are different with white copper (Fig. 8(a) showing characteristic peaks in XPS images, i.e., copper, nickel, fluorine, silicon, carbon, oxygen) and 95Ag (Fig. 8(b) showing characteristic peaks in XPS images, i.e., silver and fluorine, silicon, carbon, oxygen), the coating materials is the same of suspension, thus presenting similarity in micro-morphology of coatings (Fig. 8(c) and (d) of SEM images).

Sample 2, as roughened Miao silver by sandpaper, enjoy water CA of 64°, decreased by 5° from 69° (Sample 1), getting more hydrophilic, which Wenzel Equation reveals. Followed by coating hydrophobic materials, fluorinated modified silane or SiO_2_-fluorinated modified silane suspension, water CA increases obviously, from hydrophilic to hydrophobic, compositions interacting with microstructure, indicating their synergy. Therefore, difference in microstructure and compositions from sample 1 to 10 as well as, directly results in difference in water CA, thus CAH, solid-liquid interface tension, adhesion work and spreading coefficient. Based on their change rule, we can find main factors, quantifying relationships and related proof.

## Method validation

The results obtained include theoretical computation and corresponding experiment test, of which the main property parameters are CA together CAH, roughness and micro-morphology. Validation of preparation and theoretical modeling is indicated as the following.

Firstly, the consistent of preparation with modeling get to be confirmed, implying the reasonability of bio-simulation. From appearance of model, it is totally inclined to like the microstructure of butterfly’s swings. Experimentally, by coarse/fine sand-grinding, signs of grinding in microscale are used to simulate the first-step large semicircle microstructure with diameter of structure unit in 135 μm or so, and the second-step small semicircle microstructure with diameter of structure unit in 15±0.3μm(Fig. 8(c) together inset). Referring to the ratio of micro/nano scale of 100 from lotus leaves, we set this ratio from 50 to 100 and keep the morphology of surface with large microsemicircle encircled by small microsemicircle along the semi-circular path in cross section as substrate, followed by nano SiO_2_ grain covering it (Fig. 8(c) and (d) flower-shaped or chorionic villous structure). Therefore the morphology of surface model will not change with R/r of ratio; the consistence of model and preparation gets to be verified. Although the preparation, with our simple method, is hard to satisfy the requirement of theoretical modeling, even possibly having obvious difference between them, fortunately, the test results are largely close to computation, taking sample 8, its initial arithmetic mean roughness (one-dimension linearity) from sandpaper-grinding is about 1.868 (Table 1), if transferred to area roughness used for Wenzel equation, 3.49 or so; after coating suspension, such roughness value being over 4 is possible; the obtained CA together CAH of 162°and 4° (165°minus 161°) respectively (Fig. 5(e)); corresponding computation results are: given the roughness between 4.85 to 4.94, CA minimum of 166°, CAH from 1° to 3.5°; having no obvious difference between them, namely, quantitatively fitting. Using software carried by CA measurement instrument (SDC-350) to indirectly measure the adhesion work, the result shows us that, with increase of the CA, the tension of solid-liquid interface also increases with it, implying obvious phase separation; inversely, the *w_a_* together spreading coefficient of *S_l/s_* decreases (Table 1), indicating inhibition of wetting. Meanwhile, the computation gives the same result as well (Fig. 6(b_2_)-(e_2_)). All in all, both experiment and computation fit with each other, quantitatively or qualitatively, despite that the experiment technique is simple and economic.

Secondly, experiment method designed by us has self speciality, integrating the traditional etching with coating, but further developing, winning the success in fabrication of SHS, especially based on Miao silver. Currently the academic community uniformly thinks that the SHS are typically fabricated in one of two ways: (i) creating roughness on an inherently hydrophobic surface; or (ii) modifying a rough surface with a low surface energy coating [12,13]. Compared with already used chemical etching and coatings technique, i.e., spin/spray/dip/ coating [[33], [34], [35], [36], [37]], our creation mainly presents three aspects (advantage):

1. Using etching in physical rather than chemical mode to obtain a high roughness surface, sandpaper-grinding similar to metal-drawing on Miao silver surface not changing the chemical compositions of surface.
2. Remoulding coatings’ chemical compositions by doping SiO_2_ nano-particles to further increase the surface roughness in nano-scale [25].
3. Environmentally friendly, execution of the method does not bring in environmental issue due to etching in physical mode and avoiding toxic chemicals in selection of hydrophobic coatings for surface modification. Therefore, such a two-step method of integrating physical sand-grinding with doping hydrophilic coatings as basic technical skill is the core of study, being practical and easy to develop on an industrial scale. Here, note that, although the particles appear hydrophilic, after brush Coating, it is enclosed by hydrophobic fluorinated modified silane, not affecting the hydrophobicity of surface, only enhancing the roughness of surface, thus selected nano-particles for doping is diversified according to application requirement.

For progress to understand of modeling and preparation together comparison with different method, referring to related literature, we managed to sort series of classical techniques, especially related to etching (Table 2) [12].

**Table 2:** Comparison of different techniques for superhydrophobic surfaces.

Technique	Overview of method	Attendant advantages	Attendant disadvantages
Sand-paper-grinding [25]	Similar to metal drawing, firstly to form rough surface (up-down) then to spray/brush nano-coating with low surface energy(down-up)	Simple, fast, cost-efficient, no specific requirement for device, scalable and easy to be industrialized	Adhesion between coatings and substrate needs to be improved, same to stability
Spin, spray, dip coating [[38], [39], [40]]	Down-up to supply coatings to substrate	Fast, cost-efficient, scalable, but process not being simple for dip coating, and damage to wide range of materials	Poor coatings stability and uniformity being hard to meet application requirement and not environmentally friendly
Chemical etching [41]	Up-down to form rough surface on the substrate then down-up to modify it with hydrophobic coatings	Simple, fast, cost-efficient, scalable	Hard to avoid use of toxic reagents, weak uniformity, mainly applicable to metals and alloys
Sol–gel [42]	A wet-chemical technique that generally comprises of two steps. The first step is the hydrolysis of nanoparticles in a solvent to produce a sol, followed by polycondensation reactions of the hydrolyzed product to form a gel(down-up)	Cost-efficient, high purity and homogeneity, size and morphology of particles being tunable, scalable and applicable to great range of materials	Slow process thus low efficiency, vulnerable to film delamination
Laser processing [43]	A laser ablation process, up-down techniques which generates structures within the scale of 10 to a few hundred micrometers, 1 to 10 µm, and <1 µm, respectively. Obtained microstructure is affected by multi-factors (scanning distance, laser fluence, pulse rep- etition rate)	Simple and relatively fast together high uniformity and precision of structures, environmentally friendly, applicable to great range of materials	Not scalable and efficient, process parameters need to be optimized
Electrodeposition [44]	An electric potential is used for the reduction of metal ions and subsequent deposition on a substrate acting as a cathode(up-down)	Cost-efficient, texture and morphology being tunable, scalable	Applicable to metals mainly
Electrospinning or Electrospraying [45]	Consisting of a feeding system, a spinneret and a pump. A high voltage applied to generate a sufficiently strong electric field. Using applied electric fields to deposit nano/microstructures(down-up)	Formation of fiber creating large surface area of homogeneity	Limited porosity control, use of organic solvents, lightly slow, limited to polymer fibers
Chemical vapor deposition [46]	A substrate is exposed to a gaseous phase of volatile precursors which react to form a film or powder layer on the solid substrate(down-up)	Residue-free, surface homogeneity, applicable to wide range of materials	Not cost-efficient and practical for large-scale applications, high temperature/pressure, requires precise control of operating parameters thus hard to enlarge
Lithography [47]	Photoresist is spin-coated on the substrate(up-down). A photomask with a desired pattern is placed on the coated surface and exposed to UV light. The regions of negative photoresist exposed to the UV light form free radicals which cause cross-linking of polymerization upon heating. A developer solution clears away unexposed regions selectively, leaving the desired nano/micro scale structures on the substrate	Cost-efficient, controllable morphology and structure size, reusability of templates, environmentally friendly, applicable to great range of materials	Requires clean room and flat substrate, not scalable, multi-step process
Thermal deposition [48]	With thin polymer film, heated to a temperature between its softening and melting points, the desired pattern is printed on its surface by a pressure. After cooling, the polymer substrate is removed from the mold and the inverse template structure is transferred to the surface of the polymer(down-up)	Scalable, high reproducibility of film, applicable to great range of materials	Multi-step process required for large-scale production
Plasma etching [49]	Plasma species (ions, radicals, electrons, and excited molecules) are used to develop nanostructures on the surface of substrates and reduce surface energy, using particles with high kinetic energy to knock out the atomic or molecular species from the substrate(up-down)	High aspect ratio structures, applicable to great range of materials	Costly, potential toxic gas formation thus not environmentally friendly

Through comparison with existing superhydrophobic technologies, a point of view occurs to me that sand-paper-grinding surely has self advantage in requirement of device, cost-efficient and potentially large-scale production on condition that adhesion and anti-corrosion ability is improved significantly.

Thirdly, Infrared spectrum (Fig. 7(a)-(c)) and SEM images (Fig. 7(d)-(f) and Fig. 8(c)-(d)) of different samples are only used for explaining that the surface chemical compositions and micro-morphology can influence on the surface property together, implying that these factors should not be ignored on improving surface functionality[[50], [51], [52], [53]].

Summarizing the whole study process, we may generalize the route diagram as follows (Fig. 9), which presents systematicness and logic of study.

**Fig. 9:**
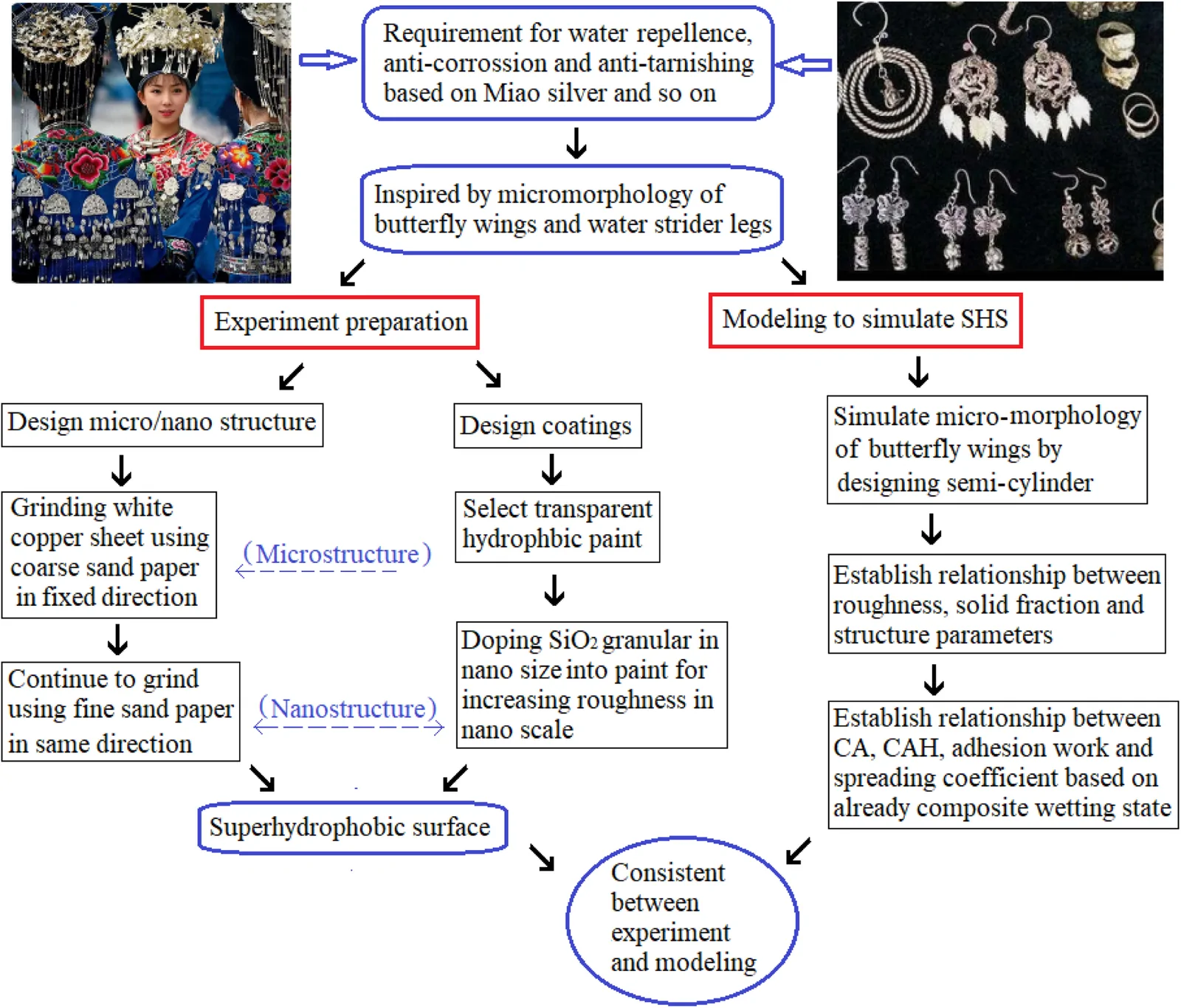
Study line for preparation and modeling of butterfly wings-simulated superhydrophobic surface.

## Limitations

None.

## Ethics statements

Our work did not involve human subjects, animal experiments and data collected from social media platforms.

## Supplementary material and/or additional information [OPTIONAL]

Video of needle dragging water droplet as “Supplementary material” has been uploaded.

## Declaration of competing interest

The authors declare that they have no known competing financial interests or personal relationships that could have appeared to influence the work reported in this paper.

## Data Availability

The data that has been used is confidential.
